# What is the impact of lateral nasal wall osteotomy depth on pterygomaxillary separation during a Le Fort I downfracture?

**DOI:** 10.4317/medoral.26939

**Published:** 2025-01-26

**Authors:** Taha Pergel, Emine Fulya Akkoyun, Guniz Kasarcioglu, Dogan Dolanmaz

**Affiliations:** 1 DDS, DS. Assistant Professor. Department of Oral and Maxillofacial Surgery, Faculty of Dentistry, Bezmialem Vakif University, Istanbul, Turkey; 2 DDS, PhD. Assistant Professor. Department of Oral and Maxillofacial Surgery, Faculty of Dentistry, Bezmialem Vakif University, Istanbul, Turkey; 3 DDS. Department of Oral and Maxillofacial Surgery, Institute of Health Sciences, Bezmialem Vakif University, Istanbul, Turkey; 4 DDS, PhD. Professor. Dean. Department of Oral and Maxillofacial Surgery, Faculty of Dentistry, Bezmialem Vakif University, Istanbul, Turkey

## Abstract

**Background:**

The study aimed to investigate the effect of customized lateral nasal wall osteotomy (LNO) on the lateral nasal wall (LNW) and pterygomaxillary junction (PMJ) separation during Le Fort I. We hypothesized that customized LNO on the LNW affect the PMJ separation type.

**Material and Methods:**

This prospective, controlled, randomized study included forty-three patients were randomly assigned to either the conventional or customized (study) osteotomy groups. In the study group, LNW depth was measured before surgery in the axial section of the CT scan, and LNO was performed at a depth of 2 mm less than the measured distance. In the conventional osteotomy group, LNO was performed at 30 mm for females and 35 mm for males. Patients with cleft lip and palate, previous orthognathic surgery, or rhinoplasty were excluded. Separation types were classified as follows: LNW types; Type1-from the osteotomy line; Type2- 2-4 mm above the osteotomy line; Type3- 4 mm or more above the osteotomy line. PMJ types; Type1-including the tuber maxilla; Type2-from the pterygomaxillary junction; Type3-including the pterygoid plates. Chi-square tests were conducted to determine whether there was a significant correlation between groups and LNW separation types, groups and PMJ separation types and groups, and LNW separation type and PMJ separation type. A *P* value of < .05 was considered statistically significant.

**Results:**

In both the conventional (*P*=0.052) and the study groups (*p*=0.828), there was no significant difference between LNW depth. Type 1 (*P*=0.0003) and Type 2 (*P*=0.0051) LNW separation types presented a significant difference between groups. A chi-square test showed a significant correlation between the surgical groups and PMJ separation patterns (*P*<0.05).

**Conclusions:**

Customized LNO optimizes the LNW and PMJ separation. Facilitates the Le Fort I surgery and decrease unintentional fracture of the PMJ.

** Key words:**Le Fort I, lateral nasal wall, pterygomaxillary junction, fracture type, arterial bleeding, orthognathic surgery.

## Introduction

The Le Fort I osteotomy is a horizontal maxillary osteotomy used to correct midface deformities, allowing three-dimensional movement. It can also facilitate surgical access for the removal of tumors or the reduction of complex midfacial fractures. The procedure was named after Rene Le Fort, who described the Le Fort I horizontal fracture type in 1901 (1).

As the procedure has continued to evolve, incorporating and recognizing the roles of hypotensive anesthesia, orthodontics, tension-free stability, and virtual surgical planning, it has become a standard, predicTable, and safe procedure.

During Le Fort I osteotomy, precise knowledge of blood vessel locations is crucial to avoid significant bleeding, particularly in the two stages most associated with arterial bleeding complications. One is lateral nasal wall osteotomy (LNO), and the other is the separation of the pterygomaxillary junction (PMJ). In particular, the descending palatine arteries are located posterior to the pyramidal processes of the palatine bone. They can be inadvertently damaged because of over-extension of the lateral nasal osteotomy. Inappropriate superior placement of the osteotome to separate the pterygomaxillary junction can interrupt the pterygopalatine fossa and damage the internal maxillary artery and its branches (2).

Lateral nasal osteotomies are performed with a small straight chisel or Neivert-Anderson single-guarded osteotome with a curved end in contact with the nasal floor. Aligning the osteotome to correspond to the divergence of the lateral nasal wall (LNW) from anterior to posterior is essential and eliminates the complications. A hard stop is encountered when the osteotome contacts the pyramidal process of the palatine bone. The nasal septum is then separated from the maxilla using a thin U-shaped osteotome by seating its curved end towards the nasal floor. Li, *et al*. (3) suggested that the LNO should not be extended to more than 30 mm in females and 35 mm in males to avoid damage to the descending palatine artery. However, they also reported the range of LNW depth between 28 and 43 mm in females and 34 to 42 mm in males. In patients with a shorter LNW depth, bleeding may occur if the osteotomy is performed at a standard depth. Therefore, performing preoperative measurements and determining the osteotomy depth in each patient would be safer.

There is no research in the literature that evaluates lateral nasal wall separation effects on the pterygomaxillary junction separation type. The study aimed to investigate the effect of customized lateral nasal wall osteotomy (LNO) on the lateral nasal wall (LNW) and pterygomaxillary junction (PMJ) separation during Le Fort I. We hypothesized that customized LNO on the LNW affect the PMJ separation type. The specific aims of the study were investigating the customized LNO effect on the PMJ separation type.

## Material and Methods

- Study Design and Sample

The authors designed and executed a prospective, controlled, randomized study approved by Bezmialem Vakif University Clinical Research Ethics Committee (2023/08/06-E.110541). The study included patients 18-50 years old who underwent orthognathic surgery at the Bezmialem Vakif University Faculty of Dentistry, Department of Oral and Maxillofacial Surgery. The procedures used in this study adhere to the tenets of the Declaration of Helsinki. The inclusion criteria for the study were patients who required orthognathic surgery to treat malocclusion, sleep apnea, or jaw asymmetry. Patients with cleft lip and palate, previous orthognathic surgery, or rhinoplasty were excluded.

- Study Variables

Predictor Variables: The primary predictor variable was to investigate the customized LNO. Informed consent was obtained from all patients. All patients received a detailed explanation of the surgical procedure and potential adverse events and underwent a preoperative anesthesia evaluation. A total of 43 patients (N=43) were included in the study and computer-generated random assigned to two groups: conventional (*n*=21) or customized osteotomy (study) (*n*=22) groups. In the study group patients LNO was performed 2 mm less than the measured distance from the piriform aperture to descending palatine artery. In the conventional group patients LNO was performed 30 mm in females and 35 mm for males (3).

**Figure 1:**
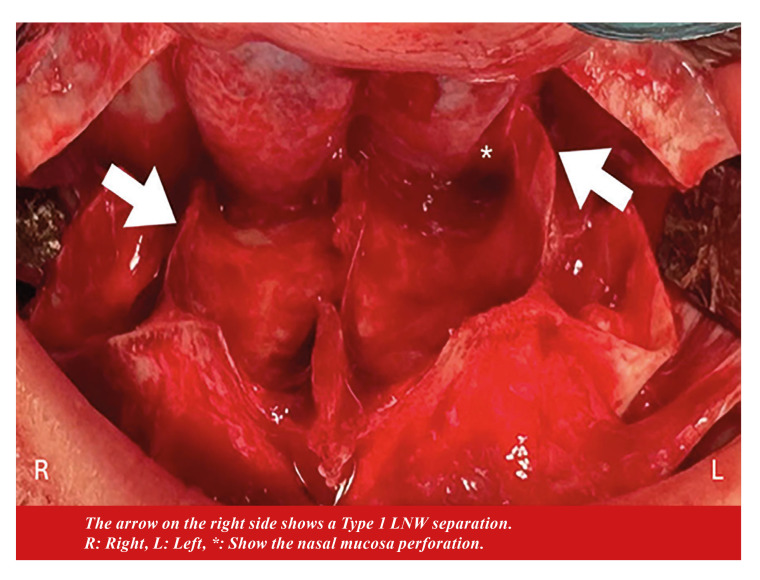
Assessment of the lateral nasal wall (LNW) separation type. The arrow on the left side shows a Type 2 LNW separation.

Other Variables: The primary outcome variable was to evaluate PMJ separation type according to customized LNO. When the maxilla was down-fractured, LNW and the pterygomaxillary separation types were assessed intraoperatively (Table 1) (Fig. 1). This is because when the maxilla is down-fractured, the PMJ fracture line is within the surgical field of view in type 1 and type 2 fractures. Fractures that are not within the field of view were classified as type 3. Each lateral nasal wall and pterygomaxillary junction were evaluated independently as a separate sample.

The secondary outcome variable was to evaluate LNW Length, PMJ thickness and width. Patients underwent a CT scan in the supine position using a 64-detector row CT scanner (Philips Ingenuity 128, Holland). The scanning area covered the space between the mandible and the cranium base. The scanning parameters were set to 120 kVp, 450 mAs, 0.5 mm slice thickness, and 0.3 mm reconstruction interval.

**Figure 2:**
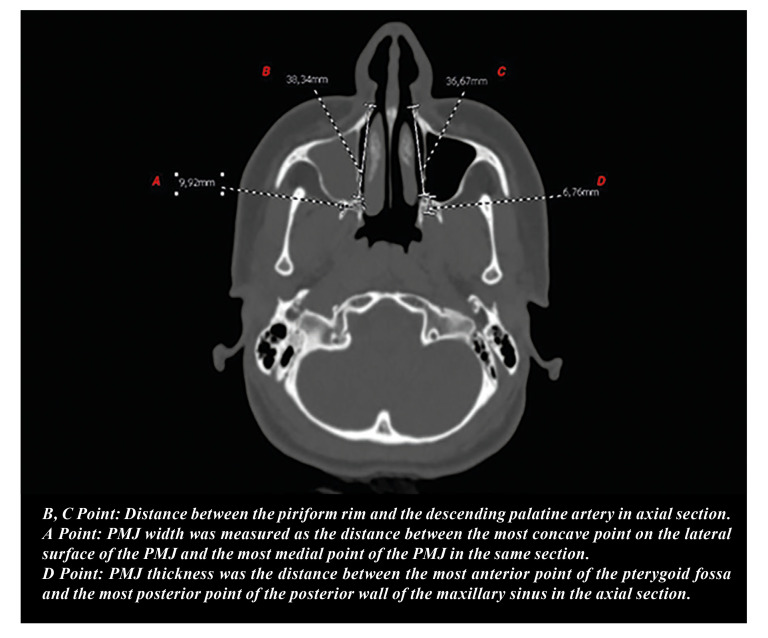
Lateral nasal wall (LNW) depth (B,C), pterygomaxillary junction (PMJ) thickness (D) and PMJ width (A) measurements in the axial CT section.

The computed tomography (CT) scans were assessed prior to surgery. The CT images were aligned parallel to the Frankfort horizontal plane to ensure standard head position. The descending palatal artery within the pterygopalatine canal was identified using axial CT images. The axial section of the CT scan that fully displayed the pterygopalatine canal, about 3-5 mm above the nasal floor, was chosen for measurement. The LNW length was measured as the distance between the piriform rim and the descending palatine artery in this axial section (Fig. 2). The PMJ thickness was recorded as the distance between the most anterior point of the pterygoid fossa and the most posterior point of the posterior wall of the maxillary sinus in the same section. Lastly, the PMJ width was measured and recorded as the distance between the most concave point on the lateral surface of the PMJ and the most medial point of the PMJ in the same section (Fig. 2).

- Surgical Procedure

The same surgical team performed the osteotomies under general anesthesia with nasotracheal intubation. Hypotensive anesthesia was applied as a standard protocol in all patients. Orthognathic surgery, including Le Fort I and sagittal split ramus osteotomy, was used to correct skeletal deformities.

Bone osteotomy was initiated on the bilateral wall of the maxilla with piezosurgery after a mucosal incision and flap elevation. The surgical steps were performed in the following order: separation of the LNW, separation of the nasal septum, separation of the PMJ, and down-fracture. LNO with Nievert Anderson single guarded osteotome, median septum separation with double guarded U-shaped osteotome, and pterygomaxillary junction separation with curved osteotome were performed before the down-fracture. A curved osteotome and a mallet were used in each case to separate the pterygomaxillary junction. The down-fracture of the maxilla was performed using a hook and a bone spreader in all patients. The maxilla was stabilized in its new position by four L-shaped osteosynthesis mini-plates at the zygomaticomaxillary buttress and the aperture piriformis regions.

- Statistical Analysis

The data were statistically analyzed using the SPSS software, version 26.0 (SPSS Inc, Chicago, IL, USA). The level of statistical significance was set at the level of *P* < 0.05. The normality of data distribution was assessed using the Shapiro-Wilk test and histograms. The differences in lateral nasal wall depth for malocclusion types determined to show a normal distribution were examined using the Student’s t-test. Other data that did not have a normal distribution were tested using the Kruskal-Wallis test. Chi-square tests were conducted to determine whether there was a significant correlation between groups and LNW separation types, groups and PMJ separation types, the down-fracture type and groups, and LNW separation type and PMJ separation type.

## Results

Forty-three patients (conventional group *n*=21, study group *n*=22), with a mean age of 24.65 ± 6.65 years, underwent bimaxillary orthognathic surgery without any intraoperative complications, including bleeding. The distribution of anatomical and demographic variables among and within the groups were described in Table 2. There was no statistically significant difference between the groups in terms of age (*P*=0.181), sex (*P*=0.084), and deformity type (*P*=0.120). There were no statistically significant differences between the groups in terms of width of PMJ and lateral nasal wall length (*P*> 0.05). But there was a statistically significant difference between the groups in terms of thickness of PMJ (*P*=0.025). In both groups, there was no significant difference in terms of the PMJ separation type between the width and lengths of the PMJ (*P*>0.05).

There was no difference between LNW depths in two different malocclusion types in the conventional group (*P*=0.060) and the customized group (*P*=0.849).

**Figure 3:**
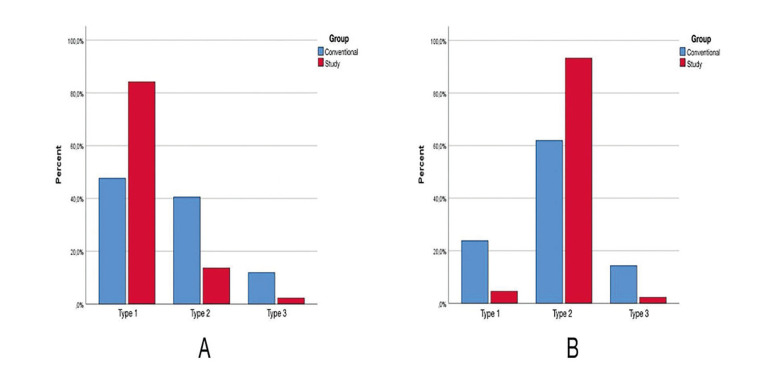
Distribution of lateral nasal wall (LNW) (A) and pterygomaxillary junction (PMJ) (B) separation patterns in the groups.

Chi-square test was performed to determine if there was a significant correlation between the surgical groups and LNW separation patterns (Fig. 3). Fisher's exact test revealed a significant correlation (χ2=12.75, *P*=0.001). The statistical differences between the groups were analyzed after Bonferroni correction (new significance level *P* <0.008) to determine which groups were responsible for this difference: Type 1 and Type 2 showed significant differences among groups (Table 3).

Chi-square test was performed to determine if there was a significant correlation between the surgical groups and PMJ separation patterns (Fig. 3). Fisher's exact test revealed a significant correlation (χ2=12.027, *P*=0.001). The statistical differences between the groups were analyzed after Bonferroni correction (new significance level *P* <0.008) to determine which groups were responsible for this difference: Type 2 showed significant difference among groups (Table 4).

**Figure 4:**
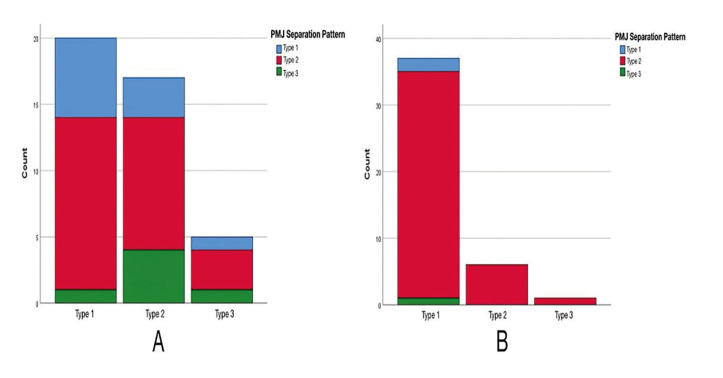
Distribution of pterygomaxillary junction (PMJ) separation patterns with respect to lateral nasal wall (LNW) separation patterns in the conventional group (A) and in the study group (B).

The distribution of LNW and PMJ separation patterns Chi-square test revealed no correlation between LNW and PMJ patterns in the conventional group (χ2=3.360, *P*=0.519) (Fig. 4). None of the patients with Type 2 and 3 LNW separation patterns in the customized osteotomy group had PMJ Type 1 and 3 separation patterns (Fig. 4). Therefore, Chi-square test was not performed in this group.

## Discussion

The aim of this study was to investigate the effect of customized lateral nasal wall osteotomy on LNW and PMJ separation during Le Fort I osteotomy. The Le Fort I osteotomy is a standard surgical procedure used to correct dentofacial deformities. The complication rates associated with this procedure range from 6.1% to 9.1% (4,5). Movement of the maxilla during down-fracture may inadvertently cause damage to the vessels and cranial nerves, leading to vision loss or life-threatening complications such as intraoperative hemorrhage, infection, and airway obstruction (6).

One major risk of this procedure is excessive bleeding due to injury of the internal maxillary artery or its branches, particularly the descending palatine artery (7,8). Over-instrumentation is the most frequent cause of descending palatal artery laceration during the lateral nasal osteotomy. In the literature, the depth of the artery is reported in various values and varies from patient to patient. Li, Meara and Alexander Jr (3) reported that the average LNW depth is 35.4 mm. Ueki, *et al*. (9) conducted a study using CT scans to determine the position of the descending palatal artery. They found that the average distance from the piriform rim to the descending palatal artery is 39.1 ± 3.8 mm on the right side and 39.4 ± 4 mm on the left side. Apinhasmit, *et al*. (10) found the lateral nasal wall length to be 34.4 ± 2.96 mm on average. In this study, females had a mean arterial distance of 32.48±3.91 mm while males had 34.27±2.97 mm, resulting in an overall length of 33.15±3.69 mm. The study of Li, Meara and Alexander Jr (3) has established the generally accepted LNO depth of 35 mm for males and 30 mm for females. However, the study also reported that the range of arterial length for females varies from 28 to 42 mm, which means that the average osteotomy depth may be shorter in some patients and too long for others. If the LNO depth is applied shorter than required, the LNW separation in the posterior region may extend higher to the anterior cranial base, making mobilizing the maxilla more difficult for the expected position. In this study, LNO was performed at a customized length. In the study group, only six Type 2 LNW separations were observed. Not only short osteotomy depth but also thin or has an overly angulated LNW curvature, may cause not to separate properly during the Le Fort I surgery. In such cases, the LNW osteotome may sometimes miss or not separate it accurately. Inappropriate separation of the LNW during Le Fort I surgery may complicate the procedure, especially in maxillary impaction and advancement cases. If the LNO line is higher than it should be, it must be reduced before the maxillary impaction or advancement. During trimming, the burr may damage the descending palatal artery, leading to arterial bleeding and related complications. This problem may also increase the operation time. In both groups patients there is no arterial bleeding complications was seen, but repositioning the maxilla in desired position is more complicated in the conventional group patients. Because in conventional group trimming the remain part of the LNW take time and sometimes complicated to handle in.

Ideal PMJ separation is also a pivotal step to mobilizing the maxilla and eliminating the related complications. The PMJ consists of the posterior maxilla, the anterior part of the pterygoid process, and the pyramidal process of the palatine bone, and it houses the descending palatine vessels; the lesser and the greater palatine nerves (11). The ideal separation starts laterally in the PMJ and medially proceeds along the pterygomaxillary fissure between the maxilla and the lateral pterygoid process (12). Usually, a curved Obwegeser osteotome was used through a blind approach to the pterygomaxillary fissure. However, other approaches include the swan-neck, shark-fin osteotomes, and ultrasonic bone curette, improving safety (13-15). Successful separation of the PMJ depends on the surgeon's experience and familiarity with the technique. Precious, *et al*. (16) described down-fracturing the maxilla with finger pressure and spreader without PMJ osteotomy. Joshi, *et al*. (17) suggested using a Smith spreader and Rowe's forceps to separate the PMJ safely. Hernández-Alfaro and Guijarro-Martínez (18) described the twist technique, PMJ separation using a frontal approach and a straight osteotome driven along the standard Le Fort I horizontal osteotomy toward the PMJ. They also described the transmucosal PMJ approach using piezoelectric devices for separating the PMJ (19). In our study, PMJ separation is performed only by curved osteotome, and a hook and a spreader were used for down-fracture. After the down-fracture of the maxilla, the PMJ separation was assessed intraoperatively. PMJ separation is related to the width, thickness, and synostosis type of the PMJ and LNW separation. There was complete fusion of the entire length of the PMJ in all patients. The width of the PMJ was similar in both groups, and no statistical difference was found.

In the study group, the mean PMJ thickness was higher than that in the conventional group, and the difference was statistically significant (*P*=0.025). According to Neema et. al. (20), the overall mean width and thickness of the PMJ were 10.24 ± 1.97 mm and 6.40 ± 1.97 mm, respectively. Chin, *et al*. (21) reported a pterygomaxillary thickness of 5.1 ± 1.4 mm and a width of 9.7 ± 1.7 mm. Dadwal, *et al*. (22) found the mean width of the PMJ to be 7.8 ± 1.5 mm and the mean thickness of the PMJ 4.5 ± 1.2 mm. In our study, similar to the other studies, the width of the PMJ is 8.15 ± 1.43 mm, and the thickness of the PMJ is 3.62 ± 1.08 mm. There was a significant correlation between the surgical groups and Type 2 (*P*=0.0004) PMJ separation patterns (Table 3). Type 1 PMJ separation was more common in the conventional group than in the study group. This can be explained by the fact that the axis of rotation was more anteriorly positioned due to the shorter osteotomy depth. Therefore, when the force was applied, the fracture started in front of the PMJ.

There is no research in the literature that evaluates lateral nasal wall separation effects on the pterygomaxillary junction separation type. In our study, in the study group, except for three cases, all the other cases had Type 2 PMJ separation, so statistical analysis could not be performed. However, based on clinical observations and the high number of Type 2 PMJ separations in the study group, we concluded that the customized LNO positively affected the separation type of PMJ. During the down-fracture of the maxilla, the almost complete separation of the LNW reduced the force required for the down-fracture, facilitated the separation of the PMJ, and shifted the osteotomy line towards the PMJ.

According to study Eberlikose et. al. (23) there was a significant difference between Class I and Class III patients lateral nasal wall length (*p* < 0.001). On the other hand, this study was show that the correlation between malocclusion type (Class 2 and Class 3) and LNW depth statistically insignificant, as well as the PMJ width and also length.

It is important to note that bone density also plays a role in the separation of the LNW and the PMJ, but this study did not assess the bone density in those regions of the maxilla. Furthermore, it is worth mentioning that the amount of force applied during the down-fracture varies depending on the surgeon's dominant hand and degree of experience. In this study, the lead surgeon was right-handed and applied forces according to the overall experience. However, the study did not evaluate the down-fracture forces applied to the maxilla. As a further limitation of the study, there are two LNWs and deviation on one side may affect the separation type on the other, which could influence the outcomes.

## Conclusions

During Le Fort I surgery, the separation of the LNW and PMJ is a critical step. However, this step is closely related to complications such as bleeding. Recent studies have reported more accurate outcomes with the use of customized applications. The results of our study indicate that using customized LNO simplifies the surgery and optimizes the LNW separation. Moreover, PMJ separation is better in patients who undergo customized LNO. Ideal separations of the LNWs shift the rotation axis towards the PMJ, and separation of the PMJ goes through the curved osteotomy line on the posterior maxillary wall. Further studies are needed to investigate the effects of the down-fracture forces and the bone density values on separation patterns.

## Figures and Tables

**Table 1:** Descriptions for the separation types of lateral nasal wall (LNW) and pterygomaxillary junction (PMJ).

Separation types		Description
Lateral Nasal Wall	Type 1	Separation from the osteotomy line
	Type 2	Separation 2-4 mm above the osteotomy line
	Type 3	Separation 4 mm or more above the osteotomy line
Pterygomaxillary Junction	Type 1	Separation including the tuber maxilla
	Type 2	Separation from the pterygomaxillary junction
	Type 3	Separation including the pterygoid plates

**Table 2:** Distribution of anatomical and demographic variables among groups.

Variables		Groups			*P*
		Conventional	Study	Overall	
Demographic variables	Age (mean ± SD, year)	24.05 ± 6.37	25.31 ± 7.00	24.65 ± 6.65	0.181
	Sex (male/female)	20 / 22	12 / 32	32 / 54	0.084
Deformity type	Class 2	10	4	14	0.120
	Class 3	32	40	72	-
Anatomical variables	PMJ thickness (mean ± SD, mm)	3.36 ± 0.86	3.86 ± 1.24	3.62 ± 1.08	0.025
	PMJ width (mean ± SD, mm)	8.10 ± 1.26	8.19 ± 1.59	8.15 ± 1.43	0.760
	Lateral nasal wall depth (mean ± SD, mm)	33.10 ± 3.38	33.18 ± 4.03	33.15 ± 3.69	0.917

**Table 3:** Distribution of lateral nasal wall (LNW) separation types among groups (new significance level was *P* <0.008 after bonferroni correction).

LNW separation types		Groups		Total
		Conventional	Study	
Type 1	Count	20	37	57
	Expected count	27.8	29.2	57
	% within grup	47.60%	84.10%	66.30%
	Adjusted Residual	-3.6	3.6	-
	Adjusted P value	0.0003		
Type 2	Count	17	6	23
	Expected count	11.2	11.8	23
	% within grup	40.50%	13.60%	26.70%
	Adjusted Residual	2.8	-2.8	-
	Adjusted P value	0.0051		
Type 3	Count	5	1	6
	Expected count	2.9	3.1	6
	% within grup	11.90%	2.30%	7.00%
	Adjusted Residual	1.8	-1.8	-
	Adjusted P value	0.0719		
Total	Count	42	44	86
	Expected count	42	44	86
	% within grup	100.00%	100.00%	100.00%

**Table 4:** Distribution of pterygomaxillary junction (PMJ) separation types among groups (new significance level was *P* <0.008 after bonferroni correction).

PMJ separation types		Groups		Total
		Conventional	Study	
Type 1	Count	10	2	12
	Expected count	5.9	6.1	12
	% within grup	23.80%	4.50%	14.00%
	Adjusted Residual	2.6	-2.6	-
	Adjusted P value	0.0093		
Type 2	Count	26	41	67
	Expected count	32.7	34.3	67
	% within grup	61.90%	93.20%	77.90%
	Adjusted Residual	-3.5	3.5	-
	Adjusted P value	0.0004		
Type 3	Count	6	1	7
	Expected count	3.4	3.6	7
	% within grup	14.30%	2.30%	8.10%
	Adjusted Residual	2	-2	-
	Adjusted P value	0.0455		
Total	Count	42	44	86
	Expected count	42	44	86
	% within grup	100.00%	100.00%	100.00%

## Data Availability

Due to privacy and ethical restrictions, the datasets generated and analyzed during the current study are not publicly available. However, they are available from the corresponding author upon reasonable request.
